# *TRIB3* promoter 33 bp VNTR is associated with the risk of cerebrovascular disease in type 2 diabetic patients

**DOI:** 10.3389/fgene.2022.916281

**Published:** 2022-08-29

**Authors:** Jiaqi Lai, Jiaying Ouyang, Weijie Lin, Mouze Liu, Yang Yang, Ruiqi Wang, Haikui Yang, Qian Meng, Jiamei Dong, Jianping Zhang, Ling Li, Fazhong He

**Affiliations:** ^1^ Department of Pharmacy, Zhuhai People’s Hospital, Zhuhai Hospital Affiliated with Jinan University, Zhuhai, China; ^2^ College of Pharmacy, Jinan University, Guangzhou, China; ^3^ Department of Pharmacy, The Second Xiangya Hospital, Central South University, Changsha, China; ^4^ Department of Pharmacy, The Fifth Affiliated Hospital of Sun Yat-sen University, Zhuhai, China

**Keywords:** TRIB3, VNTR, vascular diseases, blood pressure, antihypertensive drugs

## Abstract

Previous studies have demonstrated that *TRIB3* is closely related to insulin resistance, metabolic disorders and vascular diseases. Recently, it was reported that a 33 bp variable number of tandem repeats (VNTR) located in the *TRIB3* promoter could considerably alter its transcriptional activity. Nonetheless, whether the shift of *TRIB3* transcriptional activity has the effect of inducing diabetic vascular complications is still unclear. Therefore, in our study, we aimed to explore the relationship between the *TRIB3* 33bp VNTR and diabetic vascular complications. The *TRIB3* 33bp VNTR polymorphisms were determined by PCR and Sanger sequencing, a total of 798 eligible Chinese patients with type 2 diabetes (T2DM) were included in our study and then evaluated with clinical data. After adjusting for age, gender, BMI, smoking history, drinking history and duration of diabetes, we found that the high number of 33 bp tandem repeats (repeats>8) was significantly associated with an increase in the risk of cerebrovascular diseases compared with the low number of 33 bp tandem repeats (repeats≤6) in patients with T2DM(OR 2.66, 95% CI 1.29–5.47, *p* = 0.008). The intermediate number of 33bp tandem repeats (6 < repeat≤8) was markedly associated with a decreased risk of diabetic retinopathy compared with the low number of tandem repeats (OR 0.65, 95% CI 0.46–0.91, *p* = 0.012). Adjusting for gender, age and BMI, there was a significant difference in DBP levels among patients with the number of different 33 bp tandem repeats (Low vs. Intermediate vs. High, 81.6 ± 12.8 vs. 79.8 ± 12.4 vs. 78.7 ± 12.6 mmHg; *p* = 0.045). Subgroup analysis found that *TRIB3* VNTR was significantly correlated with the difference in systolic blood pressure (SBP) in T2DM patients taking ACEI/ARB drugs (Low vs. Intermediate vs. High, 146.27 ± 18.23 vs. 140.01 ± 19.91 vs. 140.77 ± 18.64 mmHg; *p* = 0.018). Our results indicated that *TRIB3* promoter 33bp VNTR is related to vascular diseases in T2DM patients, and may serve as a new biomarker for individualized prevention and therapy of T2DM.

## Introduction

Diabetes is a chronic disease caused by the interaction of genetic and environmental factors. According to the latest data on diabetes worldwide released by the IDF (International Diabetes Federation, 2021), from 2000 to 2021, the number of type 1 and 2 diabetic patients between the age of 20–79 rose from 151 million to 537 million. Approximately 6.7 million type 1 and 2 diabetic patients in the above age range died of diabetes and its complications in 2021. Among them, vascular complications are characterized by a high morbidity rate and are the main causes of high mortality in diabetes. The incidence rate of macrovascular and microvascular complications in patients with T2DM was about 29.7 and 52.1%, respectively (Govindarajan Venguidesvarane et al., 2020). Although the incidence rate of T2DM complications has been decreasing in recent years, there are still a large number of T2DM patients with complications (An et al., 2021). Studies have shown that the genetic polymorphism of bioactive substances can influence the risk of T2DM vascular complications, such as haptoglobin (Asleh et al., 2003) and Glutathione-S-Transferases (GST) (Sobha and Ebenezar, 2021). Therefore, the early prediction and treatment of T2DM vascular complications based on genetic factors are especially important.

VNTRs, i.e variable number of tandem repeats, are defined as stretches of DNA in which a short nucleotide sequence is repeated 20–100 times in tandem. (Lewontin and Hartl, 1991). The copy numbers of repeat elements are variable. And it may contain single nucleotide variations within the repeats or even insertions/deletions of short fragments (Marshall et al., 2021). Previous studies have shown that the VNTR polymorphism of diabetes-related genes was associated with the risk of diabetes and its complications. For instance, in IL-1RN intron 2 86bp VNTR, individuals carrying 4 repeats in both alleles were correlated with an increased risk of type 2 diabetes (Al-Safar et al., 2015). Patients with T2DM and CVD who carry the *P1 allele* of the *IL-4* VNTR polymorphism were more likely to suffer from diabetic peripheral neuropathy (Buraczynska et al., 2018). Nevertheless, a study has found that susceptibility allele A of the -23A/T INS-VNTR plays no part in the pathogenesis of diabetes in their case (Khoshroo et al., 2017). It meant that VNTR might play a seldom role in the pathogenesis of diabetes mellitus. In addition, VNTRs affect the risk of vascular disease. For example, it was proved that eNOS *intron 4 a/b* VNTR polymorphism was one of the risk factors for the development of coronary heart disease (Matyar et al., 2005). All in all, VNTR might play a role in risk and susceptibility to diabetes and vascular diseases. However, less attention has been placed on the association between VNTR and T2DM vascular complications.

Pseudokinase *TRIB3* is a stress-inducing protein that can receive various stress and metabolic signals (Hua et al., 2015). It is conserved in evolution and plays an important role in cell metabolism (Li and Hu, 2016). The 20p13-p12 chromosome region of *TRIB3* is associated with human T2DM, affects insulin signaling by inhibiting Akt phosphorylation, and is overexpressed in a mouse model of insulin resistance (Prudente et al., 2005). Many studies have shown that *TRIB3* gene variation or its altered expression plays an important role in the risk and progression of diabetes and vascular complications. For instance, the *TRIB3* R84 variant significantly increases the risk of T2DM and is related to earlier the onset age of myocardial ischemia in T2DM patients with myocardial ischemia (Prudente et al., 2005). And it can affect the risk of major macrovascular and microvascular events in T2DM patients taking different glycemic control regimens (He et al., 2016). Moreover, through the HIF1a-mediated pathway, *TRIB3* controlled critical molecular events in early diabetic retinas. *TRIB3* ablation resulted in notable retinal ganglion cell survival and functional recovery in STZ-induced mouse (Pitale et al., 2021). So, *TRIB3* may be used as a therapeutic target or indicator for diabetes and its complications. At present, a study (Ord et al., 2020)found that the 33bp VNTR of *TRIB3* promoter is one of the reasons for the difference in *TRIB3* expression among individuals. 1 to 5 copies of this repeat have been detected in the human population. The transcriptional activity increased with the increase of the 33bp repeats in the *TRIB3* promoter. To explore the influence of *TRIB3* 33bp VNTR on T2DM-related complications, our study recruited 798 Chinese T2DM patients, recorded the clinical data of each subject, and genotyped their *TRIB3* 33bp VNTR polymorphism. Through data analysis, we discovered that *TRIB3* VNTR is associated with T2DM vascular complications.

## Materials and methods

### Diagnostic and defining criteria

The diagnostic criteria for diabetes are detailed in previsous study (Yang et al., 2021). Briefly described as patients meeting the following criteria should be diagnosed with diabetes: fast plasma glucose≥7.0 mmol/L or/and 2-h plasma glucose≥11.1 mmol/L or/and HbA1c ≥ 6.5% or/and random plasma glucose≥11.1 mmol/L. Smoking/drinking history is defined as the patient with diabetes having a smoking/drinking behavior (either before or during the onset of diabetes).

According to the Chinese Guidelines for the Prevention and Treatment of Type 2 Diabetes (2020 edition), cardiovascular diseases in patients with diabetes predominantly include atherosclerotic cardiovascular disease (ASCVD) and heart failure (Chinese Diabetes Society, 2021). Diabetic cerebrovascular diseases are defined as cerebral vascular diseases induced by diabetes, resulting in intracranial large and small vessel diseases, represented by acute ischemic stroke, transient ischemic attack (TIA), and intracerebral hemorrhage (ICH) (Phipps et al., 2012; Zhou et al., 2014).

Diabetic retinopathy is a microvascular complication of diabetes that can permanently damage vision, potentially leading to blindness, and is indicated when a person with diabetes experiences sudden changes in vision or uncorrectable vision deterioration (Schorr et al., 2016; Hammes et al., 2021). The diagnostic criteria for diabetic nephropathy is albuminuria (urine albumin/creatinine ratio≥30 mg/g) and/or decreased eGFR (eGFR<60 ml/min·(1.73 m^2^)) in T2DM patients without other factors causing kidney damage (American Diabetes Association, 2017; Chinese Diabetes Society, 2021). Diabetes foot refers to patients with diabetes for the first time or with a history of diabetes, whose feet are infected, ulcerated or damaged, usually accompanied by lower limb neuropathy and/or peripheral arterial disease (Chinese Diabetes Society, 2021). Central obesity refers to a type of obesity in which fat deposition in patients is lefted on the heart and abdomen (waist circumference≥90 cm for men and 85 cm for women) (Bao et al., 2008).

### Subjects

**FIGURE 1:**
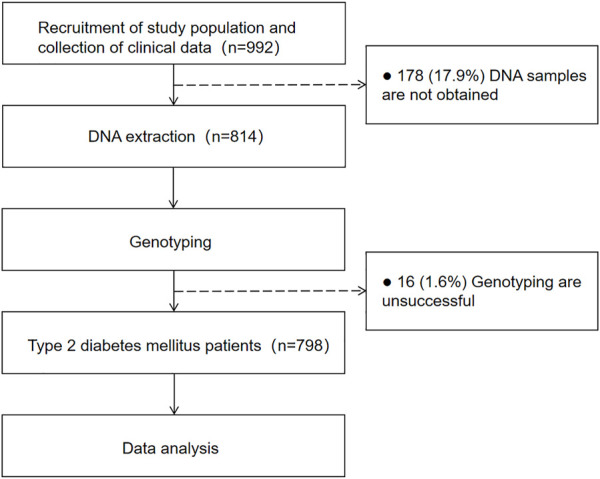
Outline of the study protocol.

This study was a retrospective study of T2DM patients in the Second Xiangya Hospital Affiliated to Central South University from 2017 to 2019. Subjects exclusion criteria were: patients who did not provide a blood sample, those who were not diagnosed with type 2 diabetes, those who refused to receive informed consent, and those who could not be genotyped in the *TRIB3* promoter 33bp VNTR. 798 subjects who were diagnosed with T2DM on admission were randomly selected, including 58.7% males and 40.6% females, aged from 17 to 88. All patients were informed and obtained written consent before the study. The clinical data, disease histories and medications of all patients were counted and analyzed. The clinical data include age, gender, BMI, waist-hip ratio, fasting blood glucose level, systolic pressure, diastolic pressure, the level of glycosylated hemoglobin (HbA1C), triglyceride, total cholesterol, HDL-cholesterol, LDL-cholesterol. Disease histories include drinking history, smoking history, cerebrovascular history, cardiovascular history and macrovascular history. In addition, the use of insulin, insulin sensitizer, β-receptor blockers, α-glucosidase inhibitor, biguanides, DPP-4 inhibitors, SGLT-2 inhibitors, GLP-1 receptor agonists, lipid-lowering drugs, calcium channel blockers, ACEI/ARB drugs and calcium dobesilate were also recorded. Among them, blood pressure, blood glucose, triglycerides, total cholesterol, low-density lipoprotein cholesterol and high-density lipoprotein cholesterol were regarded as important indicators. This study design was approved by the ethics committee in the Institute of clinical pharmacology, Central South University, and has been registered (Registration No. chictr18000 015,661, registration website: https://www.chictr.org). The outline of the study protocol can be seen in Figure 1.

### DNA extraction and genotyping

Peripheral venous blood was collected from each subject who met the inclusion criteria. Genomic DNA in blood was extracted by using E. Z.N.A.SQ blood DNA Kit II(Omega Bio-Tek Company, USA), and then stored at -80°C.Next, the *TRIB3* promoter 33bp VNTR was genotyped. The template DNA concentration in each sample was detected by Nanodrop 2000 and then amplified by polymerase chain reaction. The reaction system included template DNA 1 μL, each 1 μL of 10 μM forward and reverse primers, High fidelity enzyme MIX 10 μL, ddH2O 7 μL, The final volume was 20 μL.Primer information: the forward primer sequence is 3′-GAG​TCC​GTG​GCT​GAT​GTC​T-5′, and the reverse primer sequence is 3′-TAC​CTC​GCC​CCG​TCG​TTC-5’. The PCR reaction started at 94 °C for 5 min to denature the double-stranded DNA, followed by 35 consecutive cycles according to the three steps of denaturation at 94°C for 30s, annealing at 58°C for 30s and extension at 72°C for 30s, and finally extended at 72°C for 5 min. The length of the PCR products was shown in Supplementary Figure S1A. Then, the products were analyzed by electrophoresis in 1.5% agarose gel, and the electrophoresis results were obtained by the gel imaging system (Supplementary Figure S1B). Finally, Sanger sequencing was performed using PCR products to confirm the total number of *TRIB3* 33bp tandem repeats in both alleles of participants (Supplementary Figure S1C).

### Statistical analysis

According to the genotype frequency of the number of *TRIB3* 33bp tandem repeats in the sequencing results, combined with the results of previous studies on transcriptional activity, we divided patients into three groups based on the total number of *TRIB3* 33bp tandem repeats in both alleles: low number of 33bp tandem repeats (repeats≤6), intermediate number of 33bp tandem repeats (6 < repeats≤8) and high number of 33bp tandem repeats (repeats>8).

We calculated the sample size using sample size calculations software (Version 3.0.43) based on gene frequencies and cardiovascular event rates. The experimental data were statistically analyzed by the IBM SPSS statistics 20 program, and the results were output by five statistical methods: t-test, cross table, chi-square test, one-way ANOVA and multinomial logistic regression analysis. When calculating the mean and standard deviation of the research results, it was corrected by adding gender, age and BMI to the covariates. In order to explore the relationship between *TRIB3* promoter 33bp VNTR and common complications of T2DM, logistic regression analysis was used to calculate the odds ratio (OR) and 95% confidence interval (CI) adjusting for age, gender, BMI, smoking history, drinking history and duration of diabetes. Effect size is obtained by estimating effect size in one-way ANOVA. In addition, the picture was made by the GraphPad Prism 8 program. *p* < 0.05 was regarded as significant results.

## Result

### Clinical profile of the subject

In this study, the clinical characteristics of Chinese T2DM patients such as gender, age, smoking history and drinking history were recorded, and the number of *TRIB3* 33bp tandem repeats in each patient was measured to evaluate the relationship between *TRIB3* promoter 33bp VNTR and the clinical characteristics of subjects. The average age of participants was 58.3 years, and the standard deviation was 11.6. In the history of smoking, the proportion of patients with smoking behavior and repeats≤6 accounted for 36.3%, the proportion of patients with 6 < repeats≤8 accounted for 35.6%, and the proportion of patients with repeats>8 accounted for 43.6%. Similarly, in the drinking history, subjects with low number of 33bp tandem repeats (repeats≤6) accounted for 32.6%, subjects with intermediate number of 33bp tandem repeats (6 < repeats≤8) accounted for 29.8%, and subjects with high number of 33bp tandem repeats (repeats>8)accounted for 27.3%. The disease histories of subjects showed that patients with repeats≤6 suffered from cerebrovascular disease and cardiovascular disease, accounting for 8.10 and 13.7% respectively, 12.2 and 8.3% of patients with repeats>6,≤8 suffered from cardiovascular disease and cerebrovascular disease respectively, while the ratio of patients with repeats>8 suffering from cardiovascular and cerebrovascular disease was 11:7. The baseline clinical characteristics of subjects were shown in Table 1. Genotype frequency and allele frequency of 33bp repeats in *TRIB3* promoter were shown in Supplementary Table S1. The genotype frequency distributions were in Hardy-Weinberg equilibrium. In conclusion, no significant difference in the distribution of the three groups was found in clinical data, disease histories and drug histories.

**TABLE 1:** The characteristics of T2DM patients.

Variable	The total number of TRIB3 33 bp tandem repeats in both alleles			*p* value
	Low (repeats≤6)	Intermediate (repeats>6,≤8)	High (repeats>8)	
Male, n (%)	209 (56.9)	207 (60.7)	53 (62.4)	0.49
Age (year), mean (SD)	58.7 (11.1)	58.0 (12.1)	57.8 (11.4)	0.60
BMI (kg/m2), mean (SD)	24.3 (4.56)	24.0 (3.93)	23.9 (3.57)	0.55
Waist-hip ratio, mean (SD)				
Male	0.99	0.94	0.95	0.41
Female	0.93	0.93	0.92	0.52
GLU-60 (mmol/L), mean (SD)	11.8 (3.76)	11.8 (3.76)	12.3 (4.66)	0.75
GLU-120 (mmol/L), mean (SD)	12.6 (4.10)	12.0 (4.05)	12.0 (4.76)	0.26
Cpst-0 (pmol/L), mean (SD)	447.8 (388.4)	404.4 (316.4)	419.5 (354.3)	0.64
Cpst-60 (pmol/L), mean (SD)	649.8 (421.2)	736.2 (487.3)	826.1 (787.3)	0.087
Cpst-120 (pmol/L), mean (SD)	1,000.9 (798.6)	941.1 (739.3)	1,003.9 (851.5)	0.81
AST (IU/L), mean (SD)	22.2 (24.5)	21.8 (20.7)	20.6 (12.2)	0.92
ALT (IU/L), mean (SD)	23.3 (34.2)	21.9 (28.2)	23.6 (22.2)	0.83
BUN(mmol/L),mean (SD)	6.96 (3.46)	6.74 (2.87)	7.45 (4.30)	0.22
TBA (µmol/L), mean (SD)	5.54 (6.07)	5.91 (8.77)	5.38 (7.08)	0.79
CREA (µmol/l), mean (SD)	86.0 (79.7)	84.4 (65.1)	97.7 (131.5)	0.44
eGFR (ml/min/1.73m2), mean (SD)	95.6 (33.9)	95.2 (35.2)	92.5 (33.0)	0.74
Smoking history, n (%)	124 (36.3)	114 (35.6)	34 (43.6)	0.42
Drinking history, n (%)	111 (32.6)	95 (29.8)	21 (27.3)	0.56
History of cerebrovascular disease, n (%)	29 (8.10)	28 (8.30)	7 (8.40)	1.00
History of cardiovascular disease, n (%)	49 (13.7)	41 (12.2)	11 (13.3)	0.85
macrovascular disease, n (%)	151 (40.8)	120 (35.0)	27 (31.8)	0.14
Duration of diabetes (years), mean (SD)	9.95 (6.75)	10.87 (7.39)	10.18 (7.51)	0.23
Medical treatment				
Insulin drugs, n (%)	292 (89.0)	254 (87.9)	60 (84.5)	0.59
Insulin-sensitizing Agent, n (%)	16 (4.30)	21 (6.10)	4 (4.70)	0.56
β-receptor blocker, n (%)	58 (15.7)	61 (17.8)	22 (25.9)	0.10
Glucosidase inhibitor, n (%)	208 (56.2)	182 (53.1)	47 (55.3)	0.69
Biguanides, n (%)	171 (46.2)	158 (46.1)	41 (48.2)	0.94
DPP-4 inhibitor, n (%)	168 (45.4)	132 (38.5)	36 (42.4)	0.17
SGLT-2 inhibitor, n (%)	14 (3.80)	13 (3.80)	3 (3.50)	1.00
GLP-1 receptor agonist, n (%)	9 (2.40)	8 (2.30)	3 (3.50)	0.68
Lipid-lowering agents, n (%)	275 (74.3)	234 (68.2)	61 (71.8)	0.20
Calcium antagonists, n (%)	142 (38.4)	119 (34.7)	28 (32.9)	0.48
ACEI/ARB, n (%)	149 (40.5)	140 (40.8)	36 (42.4)	0.95
Calcium Dobesilate, n (%)	160 (43.2)	169 (49.4)	40 (47.1)	0.26

BMI, body mass index; GLU, glucose; Cpst, the secretion rate of C-peptide; AST, aspartate aminotransferase; ALT, alanine aminotransferase; BUN, blood urea nitrogen; TBA, total bile acids; CREA, creatinine; eGFR, glomerular filtration rate; ACEI, angiotensin-converting enzyme inhibitors; ARB, angiotensin receptor blockers; Low = low number of 33 bp tandem repeats(repeats≤6); Intermediate = intermediate number of 33 bp tandem repeats(6 < repeats≤8); High = high number of 33 bp tandem repeats(repeats>8); *p* < 0.05 indicates a significant statistical difference.

### Effect of the *TRIB3* promoter 33bp VNTR on common complications of T2DM

Type 2 diabetes has many complications, and patients with T2DM have a higher risk of complications. Therefore, we studied the relationship between *TRIB3* promoter 33bp VNTR and common complications of T2DM. Related data was shown in Table 2. We found that the variable number of tandem repeats of *TRIB3* promoter was significantly correlated with the risk of cerebrovascular disease and diabetic retinopathy. Among them, the high number of 33bp tandem repeats (repeats>8) markedly increased the risk of cerebrovascular disease compared with the low number of 33bp tandem repeats (repeats≤6) (OR 2.66, 95% CI 1.29–5.47, *p* = 0.008). The intermediate number of 33bp tandem repeats (6 < repeats≤8) is considerably associated with a decreased risk of diabetic retinopathy compared with the low number of 33bp tandem repeats (OR 0.65, 95% CI 0.46–0.91, *p* = 0.012). However, there were no significant correlations between this VNTR and diabetic nephropathy, diabetic foot, and central obesity.

**TABLE 2:** Effect of the *TRIB3* promoter 33 bp VNTR on common complications of T2DM.

Complications	Groups (n = 798)	Number (n, %)	OR (95%CI)	*p value
Cardiovascular disease	Low (n,%)	106 (29.0)	Ref	—
	Intermediate (n,%)	78 (22.9)	0.74 (0.50,1.10)	0.13
	High (n,%)	20 (23.5)	0.72 (0.38,1.39)	0.33

Cerebrovascular diseases	Low (n,%)	35 (9.5)	Ref	—
	Intermediate (n,%)	42 (12.2)	1.52 (0.90, 2.57)	0.12
	High (n,%)	18 (21.2)	2.66 (1.29, 5.47)	**0.008**

Diabetic retinopathy	Low (n,%)	165 (45.0)	Ref	—
	Intermediate (n,%)	125 (36.7)	0.65 (0.46, 0.91)	**0.012**
	High (n,%)	34 (40.0)	0.80 (0.46,1.37)	0.41

Diabetic nephropathy	Low (n,%)	139 (37.6)	Ref	—
	Intermediate (n,%)	124 (36.2)	0.81 (0.57, 1.14)	0.22
	High (n,%)	27 (31.8)	0.69 (0.39, 1.22)	0.20

Diabetic foot	Low (n,%)	34 (9.2)	Ref	—
	Intermediate (n,%)	33 (9.6)	1.08 (0.63, 1.86)	0.78
	High (n,%)	7 (8.2)	1.05 (0.44, 2.54)	0.91

Central obesity	Low (n,%)	185 (50.0)	Ref	—
	Intermediate (n,%)	151 (44.0)	0.69 (0.46,1.04)	0.075
	High (n,%)	32 (37.6)	0.54 (0.28, 1.05)	0.070

Low = low number of 33 bp tandem repeats(repeats≤6); Intermediate = intermediate number of 33 bp tandem repeats(6 < repeats≤8); High = high number of 33 bp tandem repeats(repeats>8); Ref.:reference; *p value was adjusted for age, gender, BMI, smoking history, drinking history and duration of diabetes, *p* < 0.05 indicates a significant statistical difference. The bold values means that the value reaches a significant difference.

### Relationship between *TRIB3* promoter 33bp VNTR and blood pressure, lipids and glucose in T2DM patients

In addition, we analyzed the relationship between *TRIB3* promoter 33bp VNTR and subjects’ blood pressure, blood lipid and blood glucose. The study showed that the 33bp VNTR was related to the blood pressure of patients, and there was a significant difference in diastolic blood pressure among patients with the total number of diverse *TRIB3* 33bp tandem repeats (*p* = 0.045). The diastolic blood pressure of patients with repeats≤6 was 81.6 ± 12.8 mmHg, that of patients with 6 < repeats≤8 was 79.8 ± 12.4 mmHg, and that of patients with repeats>8 was 78.7 ± 12.6 mmHg. However, there were no significant differences in glycosylated hemoglobin, systolic blood pressure, triglycerides, total cholesterol, low-density lipoprotein cholesterol, high-density lipoprotein cholesterol and fasting blood glucose among the total number of distinct 33bp tandem repeats (Table 3).

**TABLE 3:** Relationship between *TRIB3* promoter 33 bp VNTR and blood pressure, lipids and glucose in T2DM patients.

Variable	The total number of *TRIB3* 33bp tandem repeats in both alleles			*p value	Effect size
	Low (repeats≤6)	Intermediate (repeats>6,≤8)	High (repeats>8)		
Fasting blood glucose (mmol/l), mean (SD)	7.68 (2.89)	7.45 (2.72)	7.33 (3.08)	0.61	0.001
HbA1c (%),mean (SD)	8.63 (2.03)	8.62 (2.00)	8.23 (1.88)	0.21	0.004
SBP (mmHg), mean (SD)	138.1 (19.8)	134.3 (19.9)	135.3 (20.6)	0.078	0.007
DBP (mmHg), mean (SD)	81.6 (12.8)	79.8 (12.4)	78.7 (12.6)	**0.045**	0.008
TG (mmol/L), mean (SD)	2.05 (2.08)	2.02 (1.90)	2.03 (1.41)	0.99	0
CHOL (mmol/L), mean (SD)	4.37 (1.21)	4.35 (1.20)	4.31 (1.14)	0.99	0
LDL-CH (mmol/L), mean (SD)	2.73 (1.02)	2.77 (1.03)	2.72 (0.96)	0.77	0.001
HDL-CH (mmol/L), mean (SD)	1.04 (0.30)	1.04 (0.29)	1.02 (0.30)	0.92	0

HbA1c, Hemoglobin A_1c_; SBP, systolic blood pressure; DBP, diastolic blood pressure; TG, triglyceride; CHOL, cholesterol; LDL-CH, low density lipoprotein-cholesterol; HDL-CH, high density lipoprotein-cholesterol; Low = low number of 33 bp tandem repeats(repeats≤6); Intermediate = intermediate number of 33 bp tandem repeats(6 < repeats≤8); High = high number of 33 bp tandem repeats(repeats>8); *p value was adjusted for gender, age and BMI, *p* < 0.05 indicates a significant difference. The bold values means that the value reaches a significant difference.

### Effect of the *TRIB3* promoter 33bp VNTR on blood pressure of patients taking different antihypertensive drugs

**FIGURE 2:**
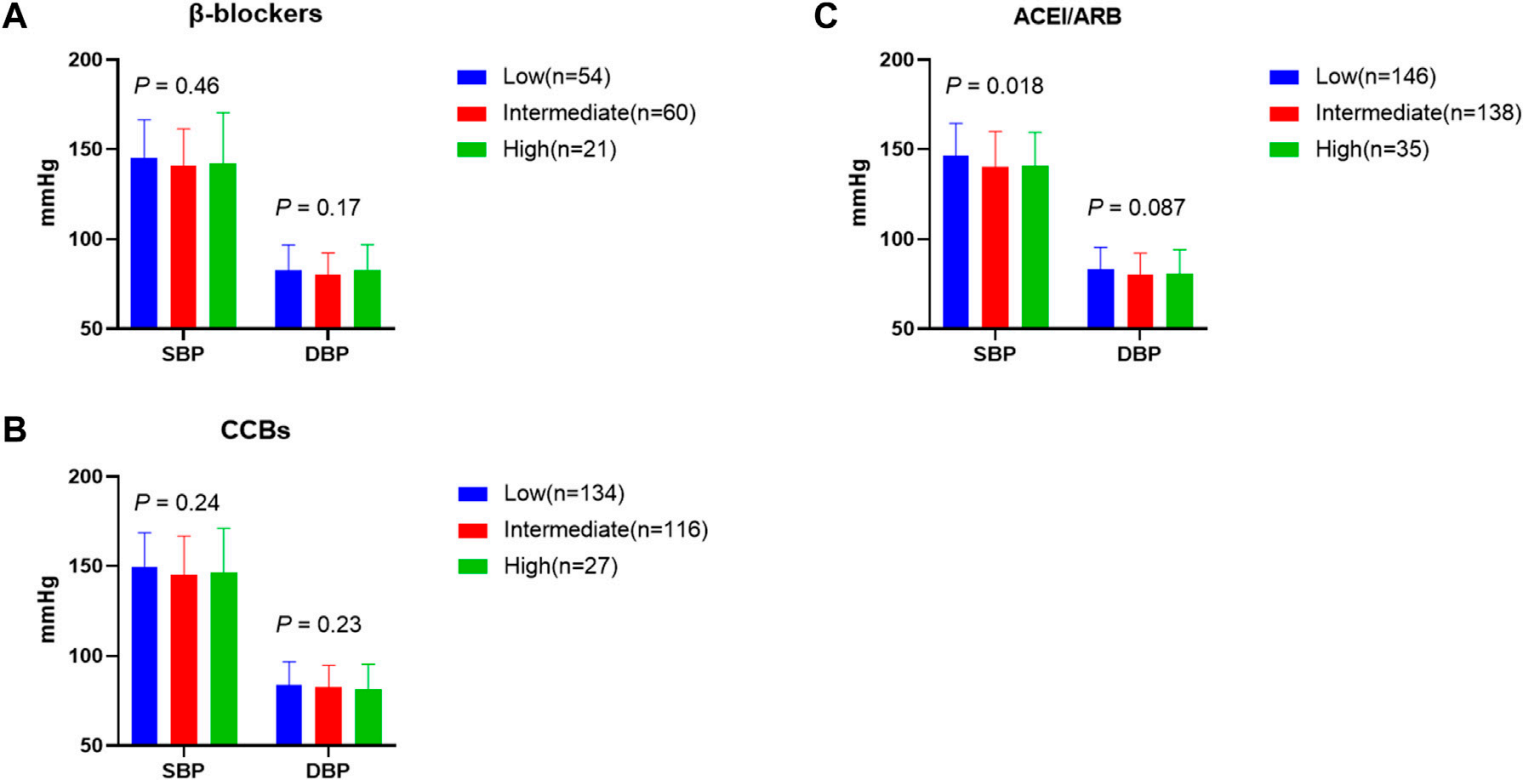
Effect of the *TRIB3* promoter 33 bp VNTR on antihypertensive drugs. Data are shown as mean ± SD, P values were calculated from one-way ANOVA with adjustments for sex, age and BMI. ACEI/ARB, angiotensin converting enzyme inhibitors/angiotensin receptor blockers; CCBs, calcium channel blockers; SBP, systolic blood pressure; DBP, diastolic blood pressure; Low means low number of 33 bp tandem repeats (repeats <6); Intermediate means intermediate number of 33 bp tandem repeats (repeats>6,≤8); High means high number of 33 bp tandem repeats (repeats>8).

Because we found that the *TRIB3* promoter 33bp VNTR was associated with the risk of cerebrovascular disease and the diastolic blood pressure of patients, we speculated that this variable number of tandem repeats might be related to the blood pressure of patients taking different antihypertensive drugs. Therefore, we simultaneously analyzed the relationship between the total number of *TRIB3* 33bp tandem repeats and the level of SBP and DBP in patients treated with β-blockers, ACEI/ARB drugs, and CCBs drugs (Figure 2). Among patients treated with ACEI/ARB drugs, the SBP of patients with the number of distinct 33bp tandem repeats had significant differences. The level of SBP in patients with the low number of 33bp tandem repeats, intermediate number of 33bp tandem repeats and high number of 33bp tandem repeats were 146.2 ± 18.23 mmHg, 140.01 ± 19.91 mmHg and 140.77 ± 18.64 mmHg, respectively; *p* = 0.018. However, this significant difference was not found in patients using β-blockers and CCBs.

## Discussion

By exploring the relationship between *TRIB3* promoter 33bp VNTR and common complications of T2DM, our study found that *TRIB3* promoter 33bp VNTR was significantly associated with the risk of cerebrovascular diseases and diabetic retinopathy for the first time. In the analysis of T2DM patients’ clinical data related to blood glucose, blood pressure, and blood lipid, we found that this VNTR was significantly correlated with the diastolic blood pressure of patients. In addition, we found that the 33bp VNTR of *TRIB3* promoter was significantly related to the systolic blood pressure of patients taking ACEI/ARB antihypertensive drugs. This study suggested that *TRIB3* promoter 33bp VNTR have biological functions.

A lot of literature has reported that *TRIB3* plays an important role in vascular disease. For example, *TRIB3* is involved in the regulation of switching phenotypes in vascular smooth muscle cells and thus has an impact on vascular disorders (Kim et al., 2014). Changes in *TRIB3* itself, such as activity and expression, can have an impact on disease progression and phenotypic alterations. In a rat model study of hypoxic pulmonary hypertension (HPH), the imbalance of *TRIB3* upregulation-mediated signaling in pulmonary artery endothelial cells played a role in the occurrence and development of HPH (Fan et al., 2020). Previous studies in type 2 diabetic rats showed that silencing the *TRIB3* gene effectively reversed aortic remodeling and improved vascular compliance (Ti et al., 2016). These studies showed that *TRIB3* variation is closely related to vascular characterization and related diseases. Recent studies showed that *TRIB3* is a significant gene linking obesity and type 2 diabetes. In obese patients with or without type 2 diabetes, the expression of *TRIB3* was higher than that in the control group. Furthermore, its expression level was significantly correlated with insulin resistance (Lee et al., 2022). In the obese state, *TRIB3* expression in skeletal muscle also exhibited the same trend and skeletal muscle-specific *TRIB3*-overexpressing transgenic mice represented impaired glucose homeostasis via disrupting insulin signaling (Kwon et al., 2018). Although *TRIB3* is implicated in both T2DM and vascular disease, the association between *TRIB3* VNTR and diabetic cerebrovascular disease has not been reported. In our findings, we found for the first time that *TRIB3* promoter 33bp VNTR is closely associated with the risk of cerebrovascular diseases in T2DM patients. Among the three groups, the high number of 33bp tandem repeats (repeats>8) significantly increased the risk of cerebrovascular disease in patients with type 2 diabetes. Given the previous study (Cao et al., 2021), we infer that the more number of 33bp repeats on the *TRIB3* promoter, the higher the transcriptional activity of *TRIB3*, resulting in *TRIB3* overexpression, and increasing the risk of vascular disease. This speculation is consistent with our observation. However, we found that the intermediate number of 33bp tandem repeats is significantly associated with a decreased risk of diabetic retinopathy. There are two reasons for this phenomenon: 1. Medication use in patients with type 2 diabetes is complicated. *TRIB3* dynamically changes with treatment and thus modifies the effect on diabetic retinopathy risk (Zhang et al., 2013). 2. Our study had a small sample size, which was insufficient to demonstrate that the intermediate number of 33bp tandem repeats reduced the risk of diabetic retinopathy.

*TRIB3* is engaged in adjusting blood pressure and its mediated AKT pathway plays an essential role in NO production and blood flow regulation (Yu et al., 2011). A previous study showed a reduction in blood pressure accompanied by a decrease in *TRIB3* expression following administration of magnololol in spontaneously hypertensive rats. When upregulating *TRIB3* expression, the Insulin-mediated NO production pathway was impaired (Liang et al., 2015). There was significant difference in DBP levels among T2DM patients with different VNTR in the study. With the increase in the number of 33bp tandem repeats, the level of DBP in patients is lower, but the effect size of this difference was small. Further verification was required in the future. Moreover, along with treatment regimens modification, the *TRIB3* expression of T2DM patients would dynamically change, which may affect blood pressure (Zhang et al., 2013). Our results suggested that *TRIB3* VNTR may influence the risk of diabetic vascular disease in T2DM patients by affecting blood pressure.

*TRIB3* is also involved in the regulation of various drug effects. Among them, there are many studies on antihypertensive drugs. For example, in a previous study on the effect of *TRIB3* rs2295490 gene polymorphism on antihypertensive drugs effectiveness, it was found that *TRIB3*(251, A > G)*AA genotype* carriers have a better antihypertensive effect than *AG/GG genotype* carriers in receiving CCBs drugs, and the levels of DBP and MAP in patients decreased significantly. In contrast, among patients taking ARBs, patients with *TRIB3*(251, A > G)*AG/GG genotype* received better antihypertensive treatment compared with *AA genotype*, in which the diastolic blood pressure changed significantly (Zhou et al., 2019). In addition, it was found that the *TRIB3* rs6037475 *CC genotype* was associated with a significant reduction in DBP levels with the extension of treatment time in patients treated with felodipine (He et al., 2020). Therefore, *TRIB3* gene mutation can affect the efficacy of antihypertensive drugs on patients. We found that the *TRIB3* promoter 33bp VNTR is significantly correlated with the level of SBP in patients taking ACEI/ARB drugs, which suggested that this VNTR may be involved in the regulatory pathway of ACEI/ARB drugs.

The diabetic macrovascular disease includes cardiovascular disease, cerebrovascular disease, and peripheral arterial disease, all of which share similar pathogenesis and risk factors. Although the *TRIB3* promoter 33bp VNTR was found to be significantly associated with cerebrovascular disease in our findings, surprisingly, this VNTR was not significantly correlated with cardiovascular disease. Moreover, when exploring whether *TRIB3* VNTR affected the blood pressure of patients taking antihypertensive drugs, we only discovered that variable numbers of tandem repeats were significantly associated with the systolic blood pressure of patients treated with ACEI/ARB medications, but not CCBs and beta-blockers. This may be due to the mechanism of action of ACEI/ARB drugs being different from that of CCBs and β-blockers, and the VNTR may only act on related proteins or genes involved in the activation pathway of ACEI/ARB drugs (Rysz et al., 2020).

Our exploratory study found that *TRIB3* promoter 33bp VNTR was associated with T2DM complicated with cerebrovascular diseases and diabetic retinopathy, which means that this variable number of tandem repeats in *TRIB3* promoter may be a new target for predicting T2DM patients with a high risk of vascular diseases in the future. So it has a certain reference value in individualized medication. Our research is a preliminary exploration. Further research is warranted to confirm whether *TRIB3* VNTR affects blood pressure, the risk of diabetic cerebrovascular disease and diabetic retinopathy and its involvement in the ACEI/ARB pathway.

## Data Availability

The original contributions presented in the study are included in the article/Supplementary Material, further inquiries can be directed to the corresponding authors.
