# Supplementary figures and images for: TRIB3 promoter 33 bp VNTR is associated with the risk of cerebrovascular disease in type 2 diabetic patients
Source: Front Genet. 2022 Aug 29;13:916281. doi: 10.3389/fgene.2022.916281 (PMC9464918; doi:10.3389/fgene.2022.916281)

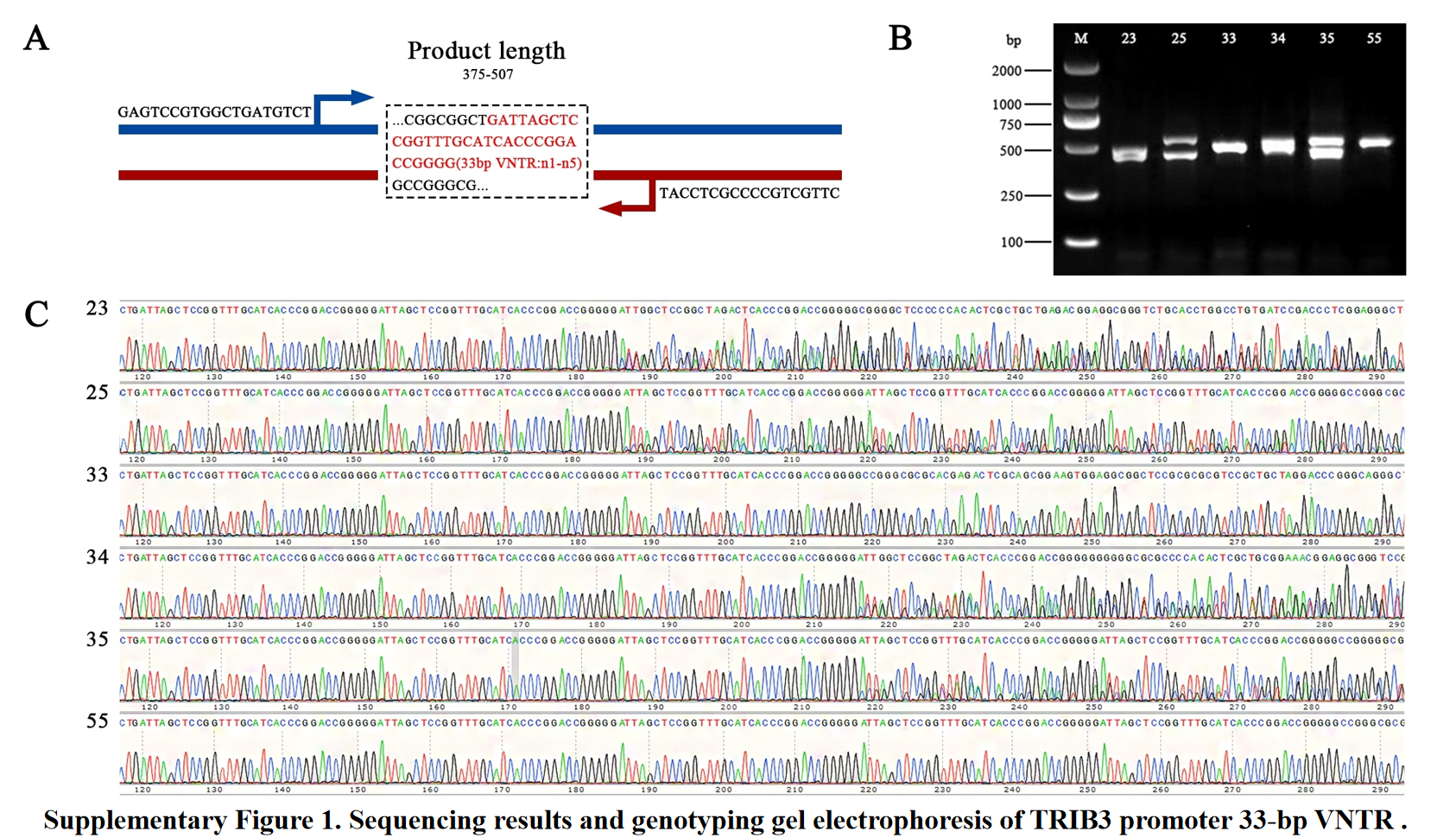

Supplement: Supplementary file 1 [file Image1.TIF]
