# Supplementary material for: TRIB3 promoter 33 bp VNTR is associated with the risk of cerebrovascular disease in type 2 diabetic patients
Source: Front Genet. 2022 Aug 29;13:916281. doi: 10.3389/fgene.2022.916281 (PMC9464918; doi:10.3389/fgene.2022.916281)
Supplement: Supplementary file 2 [file DataSheet1.PDF]

Supplementary Table 1.Genotype and allele frequencies of 33-bp repeat in TRIB3 promotor

Parameter	N（%）
**Genotypes**	**N（sum=798）**
1/3	1（0.13）
2/2	7（0.88）
2/3	75（9.4）
2/4	6（0.75）
3/3	281（35.21）
2/5	41（5.14）
3/4	40（5.01）
3/5	261（32.71）
4/4	1（0.13）
4/5	22（2.76）
5/5	63（7.89）
**Alleles**	**N（sum=1596）**
1	1（0.1）
2	136（8.5）
3	939（58.8）
4	70（4.4）
5	450（28.2）
**HWE**	**X^2=2.067, p=0.356**
